# Howe’s Acid Solution of Iron

**Published:** 1912-01

**Authors:** 


Howe's Acid Solution of Iron.—

℞ Iron sulphate, pure crystals ................... ℥ij
Nitric acid ................................ ℥j
Aqua ...................................... ℥x

Powder the iron sulphate; add the water, then the acid; bottle when the solution becomes clear. Uses: Acid-iron tonic.—*Medical Summary.*

